# Species-Specific Serological Detection for Schistosomiasis by Serine Protease Inhibitor (SERPIN) in Multiplex Assay

**DOI:** 10.1371/journal.pntd.0004021

**Published:** 2015-08-20

**Authors:** Chihiro Tanigawa, Yoshito Fujii, Masashi Miura, Samson Muuo Nzou, Anne Wanjiru Mwangi, Sachiyo Nagi, Shinjiro Hamano, Sammy M. Njenga, Evaristus Chibunna Mbanefo, Kenji Hirayama, Matilu Mwau, Satoshi Kaneko

**Affiliations:** 1 Department of Eco-Epidemiology, Institute of Tropical Medicine, Nagasaki University, Nagasaki, Japan; 2 Nagasaki University Institute of Tropical Medicine—Kenya Medical Research Institute Project, Nairobi, Kenya; 3 Centre for Infectious and Parasitic Diseases Control Research, Kenya Medical Research Institute, Busia, Kenya; 4 Production Department, Kenya Medical Research Institute, Nairobi, Kenya; 5 Department of Parasitology, Institute of Tropical Medicine, Nagasaki University, Nagasaki, Japan; 6 Eastern & Southern Africa Centre of International Parasite Control (ESACIPAC), Kenya Medical Research Institute, Nairobi, Kenya; 7 Department of Immunogenetics, Institute of Tropical Medicine, Nagasaki University, Nagasaki, Japan; 8 Consortium for National Health Research (CNHR), Nairobi, Kenya; 9 Graduate School of International Health Development, Nagasaki University, Nagasaki, Japan; George Washington University, UNITED STATES

## Abstract

**Background:**

Both *Schistosoma mansoni* and *Schistosoma haematobium* cause schistosomiasis in sub-Saharan Africa. We assessed the diagnostic value of selected *Schistosoma* antigens for the development of a multiplex serological immunoassay for sero-epidemiological surveillance.

**Methodology/Principal Findings:**

Diagnostic ability of recombinant antigens from *S*. *mansoni* and *S*. *haematobium* was assessed by Luminex multiplex immunoassay using plasma from school children in two areas of Kenya, endemic for different species of schistosomiasis. *S*. *mansoni* serine protease inhibitor (SERPIN) and Sm-RP26 showed significantly higher reactivity to patient plasma as compared to the control group. Sm-Filamin, Sm-GAPDH, Sm-GST, Sm-LAP1, Sm-LAP2, Sm-Sm31, Sm-Sm32 and Sm-Tropomyosin did not show difference in reactivity between *S*. *mansoni* infected and uninfected pupils. Sm-RP26 was cross-reactive to plasma from *S*. *haematobium* patients, whereas Sm-SERPIN was species-specific. Sh-SEPRIN was partially cross-reactive to *S*. *mansoni* infected patients. ROC analysis for Sm-RP26, Sm-SERPIN and Sh-SERPIN showed AUC values of 0.833, 0.888 and 0.947, respectively. Using Spearman’s rank correlation coefficient analysis, we also found significant positive correlation between the number of excreted eggs and median fluorescence intensity (MFI) from the multiplex immunoassays for Sm-SERPIN (ρ = 0.430, p-value = 0.003) and Sh-SERPIN (ρ = 0.433, p-value = 0.006).

**Conclusions/Significance:**

Sm-SERPIN is a promising species-specific diagnostic antigen. Sh-SEPRIN was partially cross-reactive to *S*. *mansoni* infected patients. SERPINs showed correlation with the number of excreted eggs. These indicate prospects for inclusion of SERPINs in the multiplex serological immunoassay system.

## Author Summary

More attention is now shifting towards elimination of some of the neglected tropical diseases, including schistosomiasis. Efficient diagnostics and surveillance tools are the bedrock of planning, implementation, monitoring and evaluation of such disease interventions. We had developed a multiplex immunoassay system for simultaneous detection of several pathogens in a single limited volume of human sample. To include *Schistosoma* antigen among the panel of pathogen antigens, we assessed the diagnostic value and suitability of selected *Schistosoma* antigens in multiplex format. *S*. *mansoni* serine protease inhibitor (SERPIN) and Sm-RP26 showed good diagnostic value with significant reactivity to patient plasma as compared to the control group. However, *S*. *mansoni* Filamin, GAPDH, GST, LAP1, LAP2, Sm31, Sm32 and Tropomyosin did not show disease-specific reactivity to plasma from *S*. *mansoni* infected patients. While Sm-RP26 was cross-reactive to plasma from *S*. *haematobium* patients, Sm-SERPIN showed species-specific reactivity. There was also significant positive correlation between the number of excreted eggs and fluorescence signals from the multiplex immunoassays for the SERPINs. These findings indicate potentials for utilization of SERPINs in the multiplex system.

## Introduction

Globally, more than 240 million people are still infected with schistosomiasis [1]. Over 90% of the infected people are resident in resource-limited settings in sub-Saharan Africa [2]. The next target of the current WHO roadmap for the control and elimination of schistosomiasis is to scale up mass drug administration (MDA) with Praziquantel (PZQ) [3]. Although PZQ is still efficacious in treating the disease, frequent reinfection necessitates repeated mass chemotherapy [4]. To achieve elimination, there is need for effective diagnostics to guide planning, implementation, monitoring and evaluation of the progress of the control intervention [5], and for surveillance post-elimination. Conventionally, Kato-Katz stool examination is still the gold standard for the diagnosis. However, this method is now considered relatively less sensitive than the immunological detection of circulating cathodic antigens (CCA) or circulating anodic antigens (CAA), for which specificity is still a challenge [6, 7]. Thus, there is need to continue the search for effective diagnostics with adequate specificity and sensitivity [8]. In addition to its importance in MDA based interventions, better diagnostics are required for proper assessment of the efficacy of new drugs and vaccines [9].

The distribution of schistosomiasis coincides with several other neglected tropical diseases (NTDs) and other infectious diseases, including the big three: HIV, malaria and tuberculosis [10]. Integrating the control activity of these diseases presents a unique opportunity for optimum utilization of the meagre resources for research and health care delivery, especially for the NTDs whose distributions overlap with poverty [10]. Thus, the need for the development of novel strategies to simultaneously diagnose these pathogens has been recognized. Such strategy will be potentially cost effective and more feasible given the dearth of human resources, in addition to the requirement for minimal volume of human samples [11].

Our group have been exploring strategies for reliable epidemiological surveillance for infectious diseases, especially the NTDs. In one such approach, we developed a microsphere based multiplex immunoassay system to simultaneously detect multiple infectious diseases from a single minimal volume of human sample [12]. This method is ideal in a resource-limited context and is amenable to specific epidemiological settings; depending on the prevalent etiological agents and epidemiological situations. This strategy is already deployed for screening serotypes of a single pathogen [13–16], and recently utilized by our team and others for simultaneous detection of several diseases, including NTDs [12, 17].

For an efficient multiplex detection system, careful selection of antigens to include in the multiplex immunoassay system is crucial to the efficacy of the product, and indeed is the most important aspect of the development. For application of this multiplex system in schistosomiasis endemic areas, we utilized a literature-guided approach to select a panel of 10 *Schistosoma mansoni* antigens for assessment and inclusion in the multiplex immunoassay. We identified serine protease inhibitor (SERPIN) and *S*. *mansoni* recombinant protein RP26 as promising candidates. Also, based on assessment of samples from areas of single-species and non-overlapping transmission of *S*. *mansoni* and *S*. *haematobium*, we show that SERPIN can be applied to differentially detect the *Schistosoma* species. The biological role of SERPINs and RP26, and the rationale and significance of their use for differential diagnosis of *Schistosoma* species is discussed.

## Methods

### Ethical considerations

This study was approved by the ethical review committee of Kenya Medical Research Institute, Kenya (KEMRI, SSC No. 2084) and the ethical review board of Institute of Tropical Medicine, Nagasaki University, Japan (No. 10121666 and 131219116). Written informed consent was obtained from parents/guardians and school children prior to the study.

### Sources of human plasma and sera

The samples utilized in this study were collected in a cross-sectional study conducted in Mbita and Kwale areas in Kenya between September 2011 and March 2012. The characteristics of the population and some initial analyses were detailed in a separate report [18]. Sera from amoebic liver abscess were obtained from the International Centre for Diarrheal Disease Research, Bangladesh. Diagnoses were confirmed by detection of *Entamoeba histolytica*-specific DNA in liver abscess pus specimens. Twenty *Leishmania donovani* patients’ sera were obtained from the Rajshahi Medical College in Bangladesh. Again, diagnoses were parasitologically confirmed by microscopic examination of spleen aspirates. As negative controls, sera from 32 Japanese individuals were included.

### cDNA synthesis

*S*. *mansoni* adult worms were stored in RNAlater (QIAGEN) on collection. The samples were then crushed using Tissue-Ruptor (QIAGEN). Total RNA was isolated from crushed adult worms using TRIzol plus RNA Purification kit (Ambion), according to the manufacturer’s instructions. The cDNAs of target regions were amplified by using PrimeScript High Fidelity RT-PCR Kit (Takara), according to the manufacturer’s instructions. Briefly, the first strand cDNA was synthesized from RNA with reverse transcriptase using oligo dT primer, and PCR was performed by using the reverse transcription reaction mixture as the template with a pair of specific primers for each candidate antigen. PCR products were purified using QIAEX II Gel Extraction Kit (QIAGEN). Sh-SERPIN cDNA was chemically synthesized by Integrated DNA Technologies, Inc. Introduction of G444A mutation in Sm-SERPIN was performed by overlap extension PCR. The cDNAs were then sub-cloned into pET-52b(+) vector (Novagen). Structure of the antigens and the fusion tags are summarized in Table 1.

**Table 1:** Structure of the antigens used in this study.

Antigen		N-terminus Fusion tag	Coding Region	C-terminus Fusion tag	Accession	MW (kDa)*
***S*. *mansoni***	Cathepsin B (Sm31)	MASWSHPQFEKGALEVLFQGPGYQDP	1–340	VDAAAELALVPRGSSAHHHHHHHHHH	AAA29865	44.4
	Filamin	MASWSHPQFEKGALEVLFQGPGYP	1,510–2,314	ELALVPRGSSAHHHHHHHHHH	CCD59036	92.1
	GAPDH	MASWSHPQFEKGALEVLFQGPGYQ	1–338	ELALVPRGSSAHHHHHHHHHH	P20287	41.5
	GST (Sm28)	MASWSHPQFEKGALEVLFQGPGYQDP	1–211	AAAELALVPRGSSAHHHHHHHHHH	P09792	29.4
	LAP-1	MASWSHPQFEKGALEVLFQGPGYQ	1–523	ELALVPRGSSAHHHHHHHHHH	ACQ77148	61.9
	LAP-2	MASWSHPQFEKGALEVLFQGPGYQ	1–544	ELALVPRGSSAHHHHHHHHHH	ACQ77149	64.9
	Legumain (Sm32)	MASWSHPQFEKGALEVLFQGPGYQDP	1–429	VDAAAELALVPRGSSAHHHHHHHHHH	P09841	54.8
	RP26 (Sm22.3)	MASWSHPQFEKGALEVLFQGPGYQ	20–196	ELALVPRGSSAHHHHHHHHHH	AAB81008	25.3
	SERPIN	MASWSHPQFEKGALEVLFQGPGYQDP	1–406	VDAAAELALVPRGSSAHHHHHHHHHH	CCD60071	51.8
	Tropomyosin-2	MASWSHPQFEKGALEVLFQGPGYQDP	1–284	VDAAAELALVPRGSSAHHHHHHHHHH	P42638	38.5
***S*. *haematobium***	SERPIN	MASWSHPQFEKGALEVLFQGPGYQDP	1–406	VDAAAELALVPRGSSAHHHHHHHHHH	AAA19730	51.6

The cDNAs containing the coding regions of the antigens were cloned into pET52b expression vector with StrepTagII fusion tag at N-terminus and polyhistidine tag at C-terminus.

*Predicted molecular weight

### Protein expression and purification

Recombinant antigens were expressed and purified as previously described [12]. GAPDH and Tropomyosin was located in inclusion body, whereas the other antigens were located in soluble fraction. *S*.*mansoni* soluble egg antigen (SEA) was prepared using standard methods and as previously detailed [19].

### Multiplex assay

Two antigen-coupling protocols were used in this study. For the initial experiments on *S*. *mansoni* antigens, protocol previously reported by our group was used (panel #1) [12]. Briefly, for 1.25 million MagPlex microspheres, 100μg of each antigen (except Tropomyosin) were added. Because microspheres aggregation was observed for Tropomyosin at 100μg antigen, 0.3μg of Tropomyosin was added as determined by titration experiment.

Because new protocol was released from Luminex before the subsequent experiment, the new protocol was used for the second experiment with RP26, Sm-SERPIN and Sh-SERPIN (panel #2). Binding reaction of antigen on microspheres was performed in coupling buffer (50mM MES pH 5.0) instead of PBS(-). The amount of antigens in the reaction was reduced to 25μg for 1.25 million microspheres.

### Statistics

Data analyses were performed on GraphPad Prism version 6.0. Receiver operating characteristics (ROC) analysis was performed to assess diagnostic value of antigens. Mann-Whitney tests were performed for comparison between two groups. Spearman’s rank correlation coefficient analysis was performed to assess correlation between two measured variables. Data was presented as dot plots, with lines and error bars showing the median and interquartile ranges. Statistical significance was set at p < 0.05.

## Results

### Preparation and purification of candidate antigens

**Fig 1:**
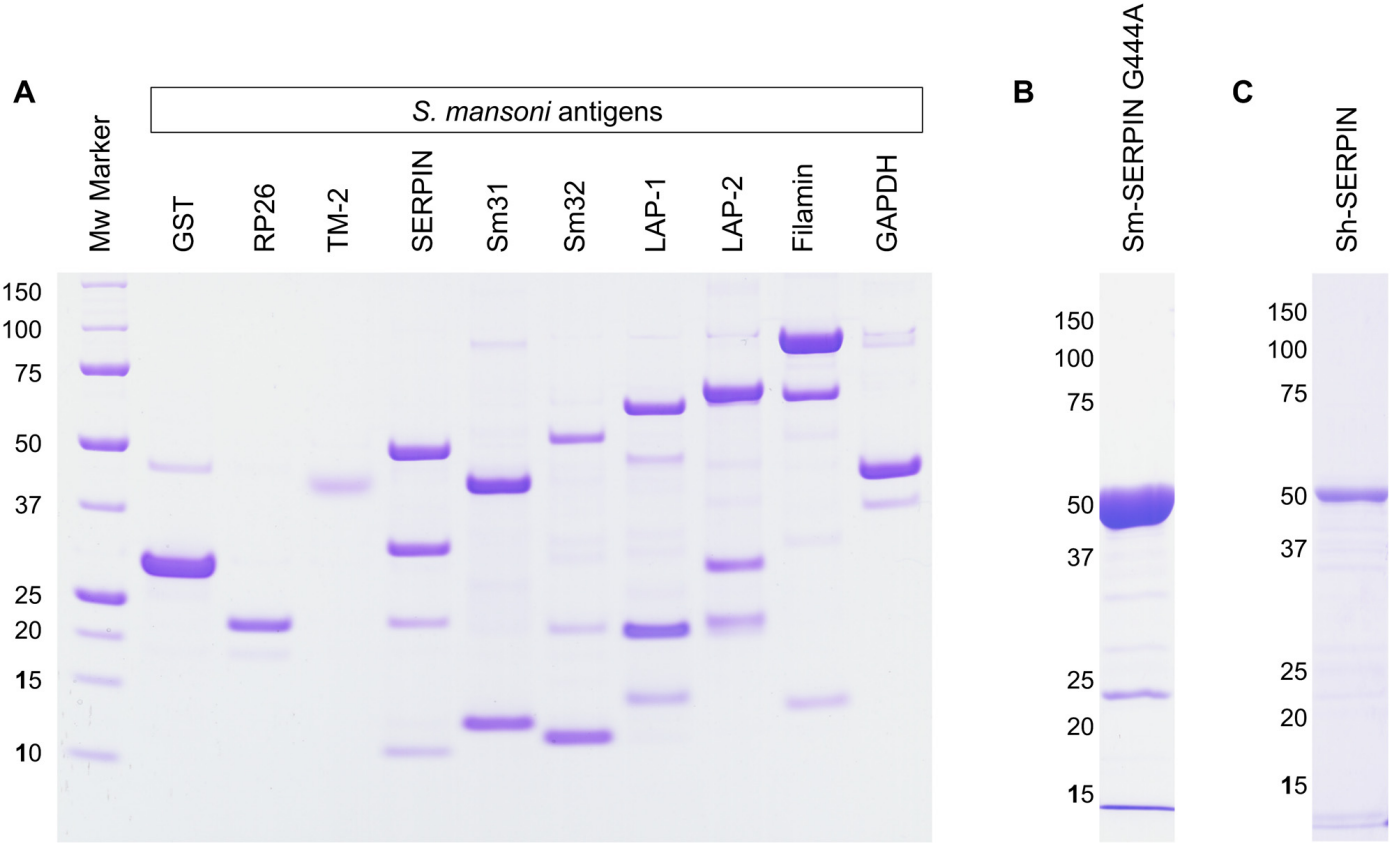
The expression and purification of selected antigens. A) SDS-PAGE of antigens for panel #1. The 10 selected antigens were expressed, purified and analysed using SDS-PAGE. B) SDS-PAGE of Sm-SERPIN G444A. For Sm-SERPIN, an unexpected shorter fragment due to transcription from prokaryotic start codon (GUG) located at nucleotide 442 was eliminated by introduction of a point mutation at nucleotide 444 of the cDNA sequence (G444A). C) SDS-PAGE of Sh-SERPIN. Sh-SERPIN was also expressed to assess species-specific identification of schistosomes by SERPINs. GST: Glutathione S-transferase, RP26: recombinant protein 26 (Sm22.3), TM-2: Tropomyosin-2, SERPIN: serine protease inhibitor, Sm31: Cathepsin-B, Sm32: Legumain, LAP-1: Leucine aminopeptidase-1, LAP-2: Leucine aminopeptidase-2, GAPDH: Glyceraldehyde 3-phosphate dehydrogenase (GAPDH).

Previously, we developed a microsphere based multiplex immunoassay system to simultaneously detect multiple infectious diseases from a single human sample [12]. To include schistosomiasis in the range of antigens in our multiplex system, we used literature-guided approach to select 10 *Schistosoma mansoni* antigens for evaluation and potential inclusion in the multiplex system. Cathepsin B (Sm31) [20], Filamin [21], Glyceraldehyde 3-phosphate dehydrogenase (GAPDH) [22], Glutathione S-transferase (GST) [23], Legumain (Sm32, Haemoglobinase) [20], RP26 (Sm22.3, LGG) [24], Tropomyosin-2 (TM-2) [25] were selected from reports on *S*. *mansoni* diagnosis. Serine protease inhibitor (Sm-SERPIN) was selected from report on *S*. *haematobium* diagnosis [26]. Leucine aminopeptidase-1 (LAP-1) [27] and Leucine aminopeptidase-2 (LAP-2) were selected from reports on *S*. *japonicum* diagnosis [27]. *S*. *haematobium* SERPIN was also included for comparison with Sm-SERPIN. Soluble egg antigen (SEA) from *S*. *mansoni* was used as control. Table 1 shows the selected antigens, their GenBank accession numbers, the N- and C-terminus fusion tags, and predicted molecular weights. The purified proteins were subjected to SDS-PAGE and protein bands of expected molecular weight were identified (Fig 1A).

### Introduction of point mutation in Sm-SERPIN cDNA

Although protein bands of expected molecular weight was identified for both Sm-SERPIN and Sh-SERPIN, we observed additional shorter fragment of about 30 kD on Sm-SERPIN lane (Fig 1A). Sequence analysis showed that this shorter fragment was a product of transcription from prokaryotic start codon (GUG) located at nucleotide 442 in the reference sequence (GenBank: CCD60071). Therefore, to compare diagnostic ability with similar quality of Sm-SERPIN and Sh-SERPIN, we introduced a point mutation at nucleotide 444 of the cDNA sequence (G444A), resulting in codon change from the prokaryotic start codon GUG to GUA. This is a silent mutation encoding for Valine. This strategy effectively reduced this extra 30 kD fragment to the minimum in Sm-SERPIN with G444A mutation (Fig 1B). The Sm-SERPIN G444A variant was therefore used in the subsequent experiments.

### Confirmation of antigen coupling

**Fig 2:**
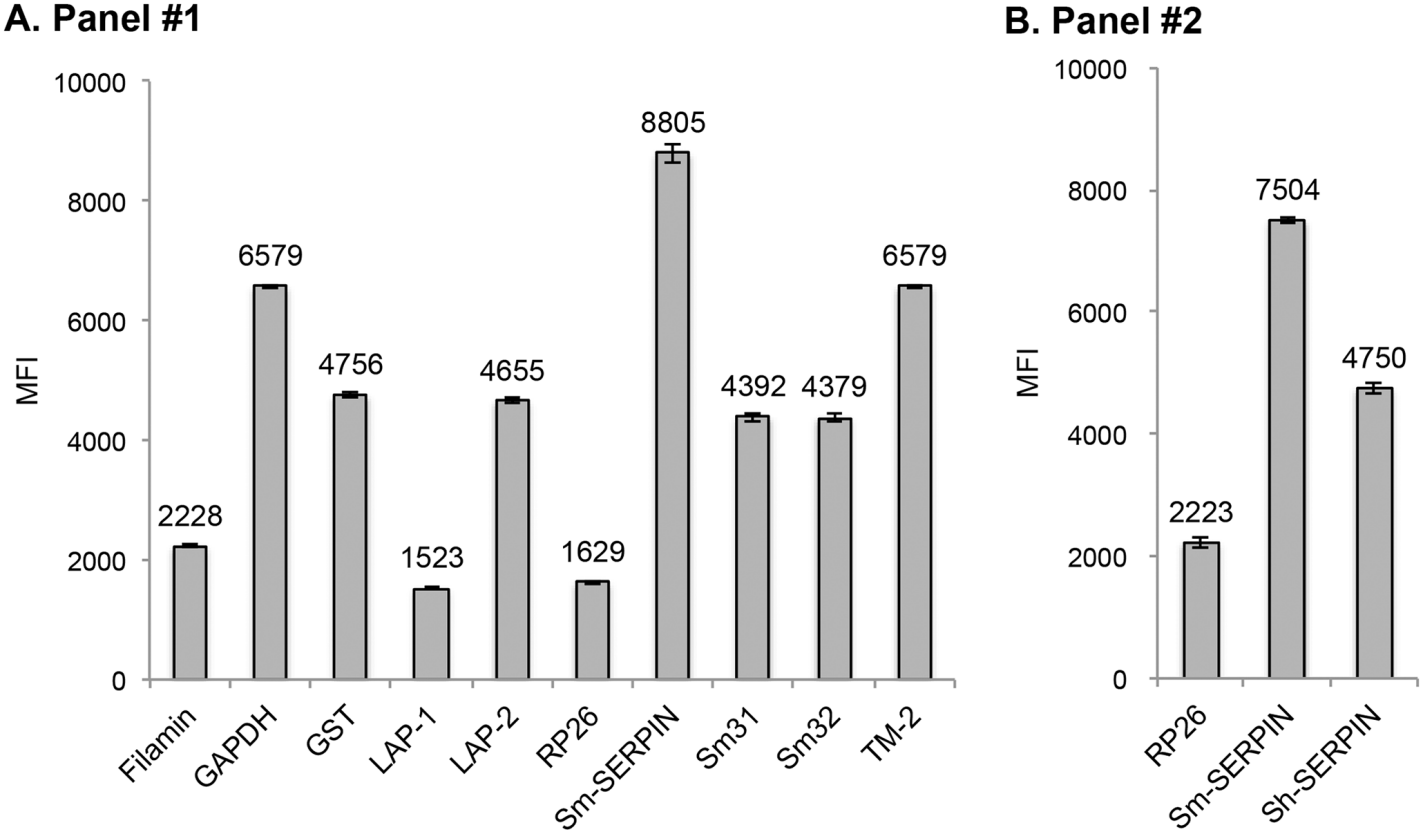
Confirmation of the efficacy of coupling of *S*. *mansoni* antigens on MagPlex microspheres. The efficacy of the coupling process for panel #1 (A) was confirmed by detection of antibodies against the poly-histidine tag on each antigen using anti-his antibody. The real measured MFI values are shown on top of bars. (B) The efficacy of the coupling process for panel #2, including RP26 and SERPINs.

To confirm success of antigen coupling to the MagPlex microsphere beads, phycoerythrin-conjugated antibody against the poly-histidine tag was used to determine the number of antigens on the microspheres. In the initial coupling beads set (Panel #1), all the antigens showed significantly high signals, indicating success of antigen coupling (Fig 2A). Relatively, SERPIN showed the highest level of success in antigen coupling (MFI = 8,805), while LAP-1 and RP26 showed the lowest level with MFIs of 1,523 and 1,629, respectively (Fig 2A). In the second coupling beads set (Panel #2), the antigens showed similar MFI values using different coupling protocol from that used for Panel #1 (Fig 2B).

### Reactivity of *Schistosoma mansoni* antigens in multiplex assay

The choice of antigens is crucial for the success of the multiplex assay development. To assess the 10 selected antigens for suitability for diagnosis of schistosomiasis in the multiplex assay, we compared the reactivity of the *S*. *mansoni* candidate antigens in multiplex format against plasma from pupils resident in two epidemiologically distinct endemic areas in Kenya. While Mbita area is endemic for *S*. *mansoni*, Kwale area is an *S*. *haematobium* endemic area. For this initial screening, twenty-six (26) plasma samples each from well characterized healthy and schistosomiasis patients from each endemic area were included in the assays and analyses. Sera from healthy Japanese volunteers were also included as negative controls from non-endemic area.

**Fig 3:**
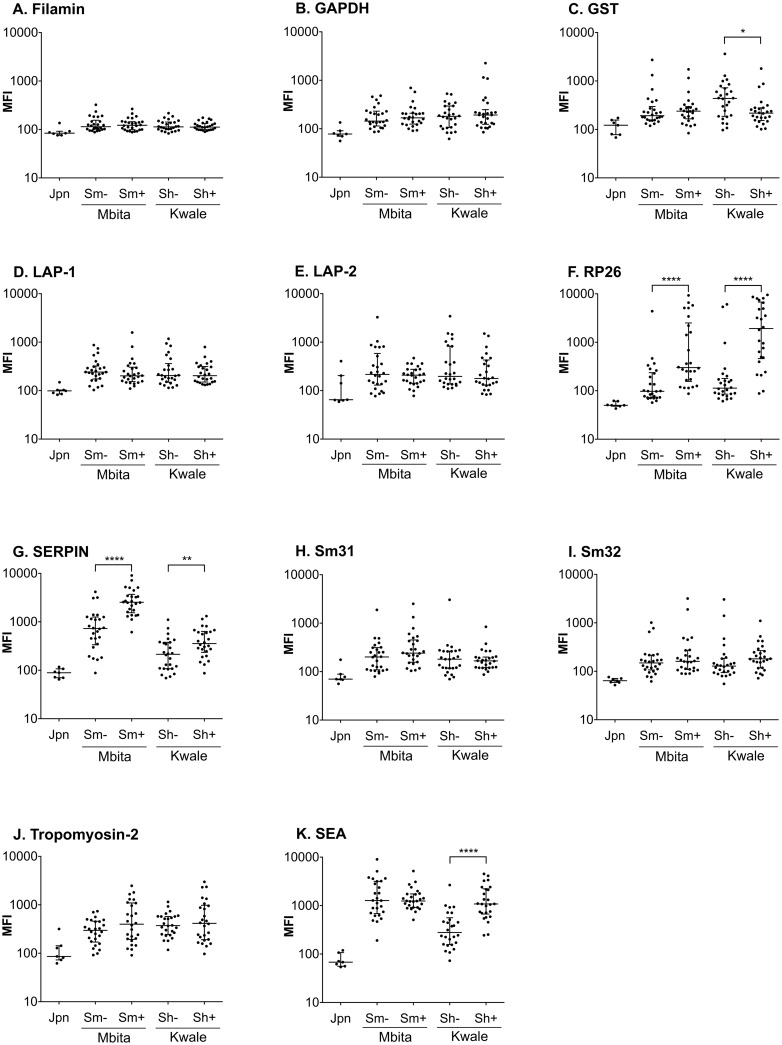
Reactivity of *Schistosoma mansoni* recombinant antigens in the multiplex immunoassay. Multiplex assays were performed using samples from *S*. *mansoni* (Mbita) and *S*. *haematobium* (Kwale) endemic areas, respectively. Negative control samples from each location were included as comparator for the diagnostic efficacy of each antigen for the two species of schistosomes. The bar lines represent the median while the error bars represent the interquartile range. Antigens used in this assay: (A) Filamin; (B) GAPDH; (C) GST; (D) LAP-1; (E) LAP-2; (F) RP26; (G) Sm-SERPIN; (H) Sm31; (I) Sm32; (J) Tropomyosin-2; (K) SEA. Statistical significance was set at p < 0.05 as depicted using asterisks: * = p < 0.05, ** = p < 0.01, *** = p < 0.001, and **** = p < 0.0001. Jpn: healthy Japanese (n = 7), Sm-: egg negative in Sm endemic area (n = 26), Sm+: *S*. *mansoni* egg positive (n = 26), Sh-: egg negative in Sh endemic area (n = 26), Sh+: *S*. *haematobium* egg positive (n = 26).

Only RP26 and Sm-SERPIN were specifically reactive to plasma from *S*. *mansoni* patients (Fig 3). The other antigens did not show disease-specific reactivity, although the reactivity to plasma from endemic areas is higher than reactivity to Japanese control as would be expected. The two antigens with disease-specific reactivity (Sm-RP26 and Sm-SERPIN) showed fluorescence signals (MFI) that are similar or higher than signals recorded from evaluation of coupling with anti-polyhistidine-tag antibody (Fig 2A). This further shows that the absence of reactivity from the other 8 antigens was not a function of number of coupled antigens. For instance, Sm-RP26 antigen with relatively low microsphere-coupled antigens based on evaluation with anti-polyhistidine-tag antibody showed significant disease-specific reactivity. Interestingly, Sm-RP26 was also reactive to plasma from *S*. *haematobium* patients (Fig 3). Our data also showed that Sm-SERPIN was efficient in species-specific differential detection of infections with *S*. *mansoni*. Also, *S*. *haematobium* SERPIN was previously reported to be species-specific [26]. The crude egg antigen (SEA) from *S*. *mansoni* showed similar MFI value between egg negative and positive in Mbita area, where *S*. *mansoni* is endemic; suggesting that some egg negative individuals may have history of previous infections. Surprisingly, SEA from *S*. *mansoni* showed disease-specific detection only for *S*. *haematobium* patients (Fig 3). Taken together, our data showed that Sm-SERPIN and Sm-RP26 are promising diagnostic candidates to be potentially included in our multiplex system. In addition, Sm-SERPIN demonstrated species-specific reactivity.

### Further characterization of the reactivity of Sm-RP26, Sm-SERPIN and Sh-SERPIN

**Fig 4:**
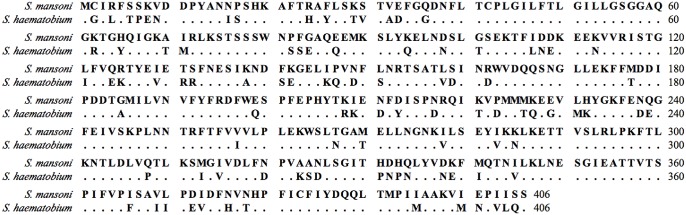
Sequence homology of amino acid for SERPINs. Dot shows homologous amino acid.

**Fig 5:**
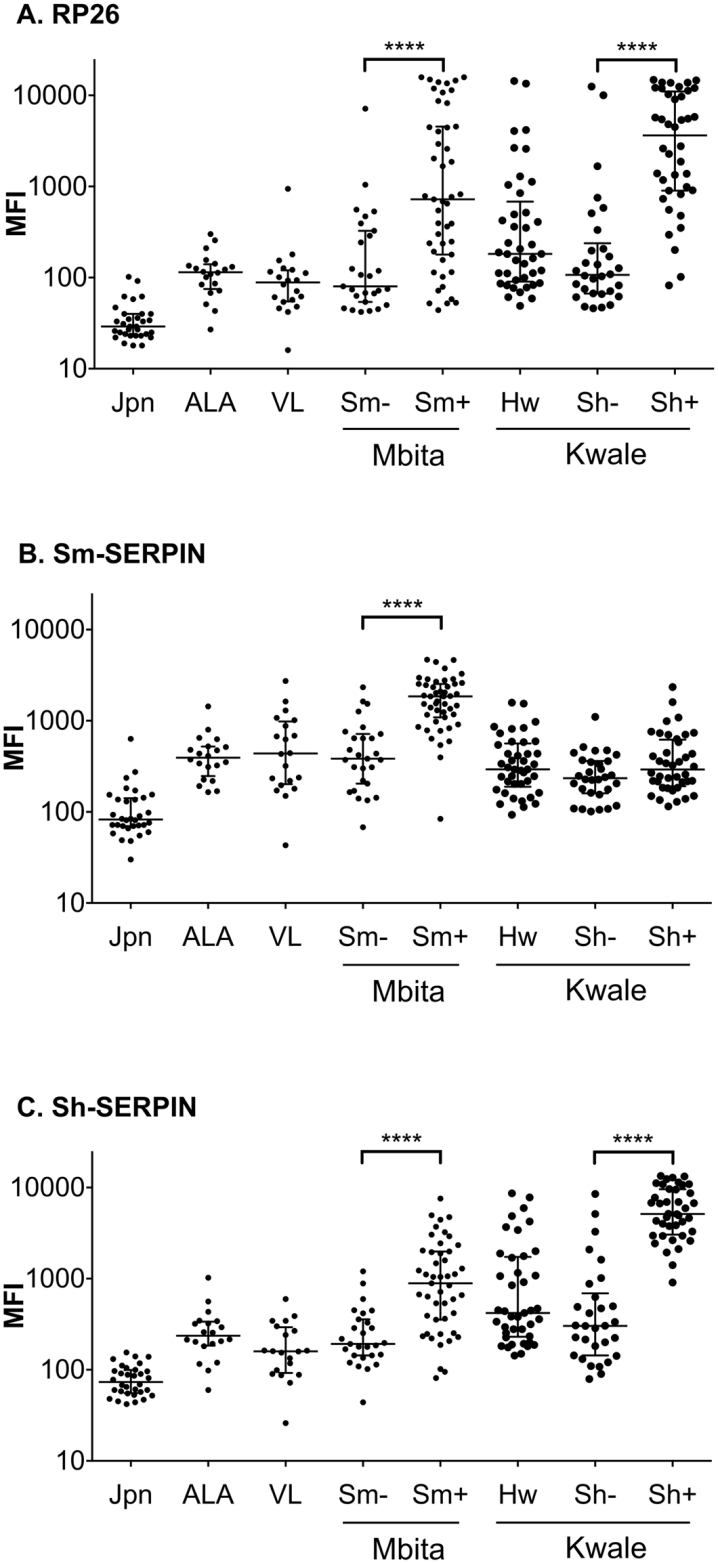
Species-specific differential detection by SERPINs. To demonstrate the species-specific diagnostic value of the SERPINS, multiplex immunoassay was performed using samples from *S*. *mansoni* (Mbita) and *S*. *haematobium* (Kwale) endemic areas, respectively. In addition, amoebic liver abscess samples, visceral leishmaniasis positive samples and hookworm positive samples were included as controls. Negative control samples from uninfected individuals from each location were also included. The bar lines represent the median while the error bars represent the interquartile range. Antigens used in this assay: (A) Sm-RP26; (B) Sm-SERPIN G444A; (C) Sh-SERPIN. Statistical significance was set at p < 0.05 as depicted using asterisks: * = p < 0.05, ** = p < 0.01, *** = p < 0.001, and **** = p < 0.0001. Jpn: healthy Japanese (n = 32), ALA: amoebic liver abscesses (n = 20), VL: visceral leishmaniasis (n = 20), Hw: Hookworm positive in Kwale (n = 41), Sm-: egg negative in Sm endemic area (n = 27), Sm+: *S*. *mansoni* egg positive (n = 47), Sh-: egg negative in Sh endemic area (n = 30), Sh+: *S*. *haematobium* egg positive (n = 40).

To further confirm the diagnostic efficacy of Sm-SERPIN and Sm-RP26 from the preceding section, we assessed a larger number of plasma for reactivity with the antigens, Sm-RP26 and Sm-SERPIN. In addition, Sh-SERPIN was included in the analysis to corroborate the species-specific diagnostic efficacy of SERPINs. Sequence homology of amino acid for the two SERPINs is presented in Fig 4. Two sequences are very similar having 76% identical amino acids. Equally, to preclude the possibility of cross reactivity with other helminths and infectious diseases, plasma from hookworm patients in Kwale area of Kenya, amoebic liver abscess (ALA) and visceral leishmaniasis (VL) patients in Bangladesh were included in the assays and analyses. Again, similar to data presented in Fig 3, Sm-RP26 was only reactive to plasma from patients with either *S*. *mansoni* or *S*. *haematobium* (Fig 5A), while Sm-SERPIN was species-specific (Fig 5B). Sh-SERPIN was partially cross-reactive to plasma from patients with *S*. *mansoni*; with median of MFI value higher in *S*. *haematobium* than in *S*. *mansoni* (Fig 5C). It was also noteworthy that cross-reaction was not observed with plasma from ALA, VL and hookworm patients (Fig 5). Our observations further provide evidence for the utility of both Sm-RP26 and SERPINs as good diagnostic antigens for schistosomiasis, in addition to the species-specific diagnostic advantage of the Sm-SERPIN.

### Validation of diagnostic performance of Sm-RP26, Sm-SERPIN and Sh-SERPIN

**Fig 6:**
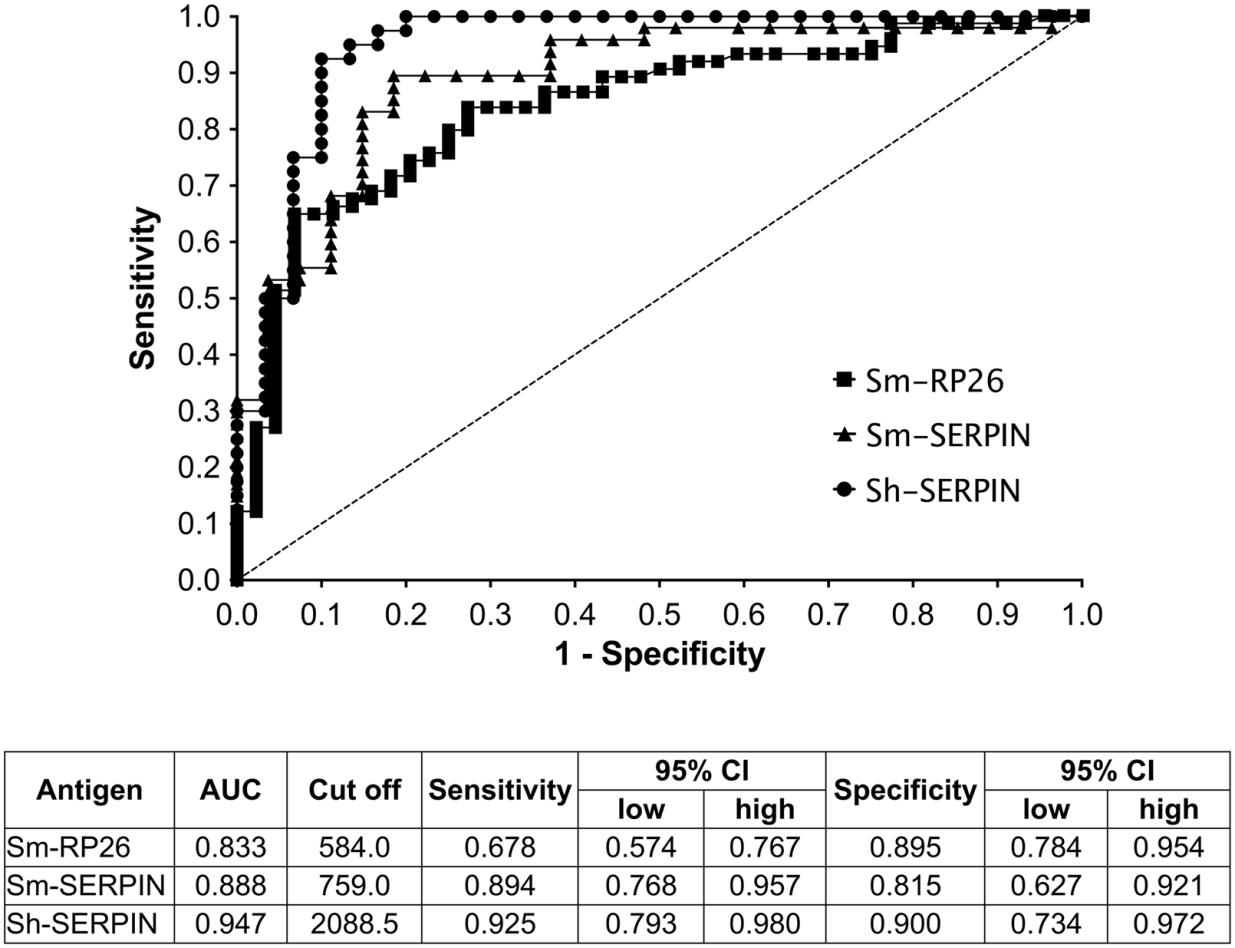
ROC analysis for RP26, Sm-SERPIN, and Sh-SERPIN. ROC analysis was performed to determine the area under the curve (AUC), sensitivity and specificity as indicators of the diagnostic value of the SERPINs and RP26. The SERPINs showed relatively better diagnostic ability than RP26.

We performed ROC analysis for further evaluation of the diagnostic performance of the SERPINs and Sm-RP26 in the multiplex assay. Sensitivity and specificity were calculated at MFI value giving maximum sensitivity plus specificity. For the ROC analysis for Sm-RP26, egg negative and positive individuals in both Mbita and Kwale were included in the analysis. For SERPINs however, data from plasma samples from the endemic area for each species were used. The ROC curve and the corresponding statistics are summarized in Fig 6. The AUC values for Sm-SERPIN (0.888) and Sh-SERPIN (0.947) were higher than AUC value for Sm-RP26 (0.833). The two SERPINs recorded sensitivity of approximately or over 90% (92.5% for Sh-SERPIN and 89.4% for Sm-SERPIN), while Sm-RP26 showed lower sensitivity of 67.8%. Sh-SERPIN was the most specific antigen with 90% specificity, followed by Sm-RP26 and Sm-SERPIN with specificities of 89.5% and 81.5%, respectively (Fig 6). Succinctly, our data indicate that the SERPINs recorded acceptable levels of sensitivity and specificity and are suitable for inclusion as diagnostic antigens in the multiplex system. The additional advantage of species-specific detection also provides further utility for these antigens.

### Correlation between number of excreted eggs and fluorescent intensity of the multiplex immunoassay

**Fig 7:**
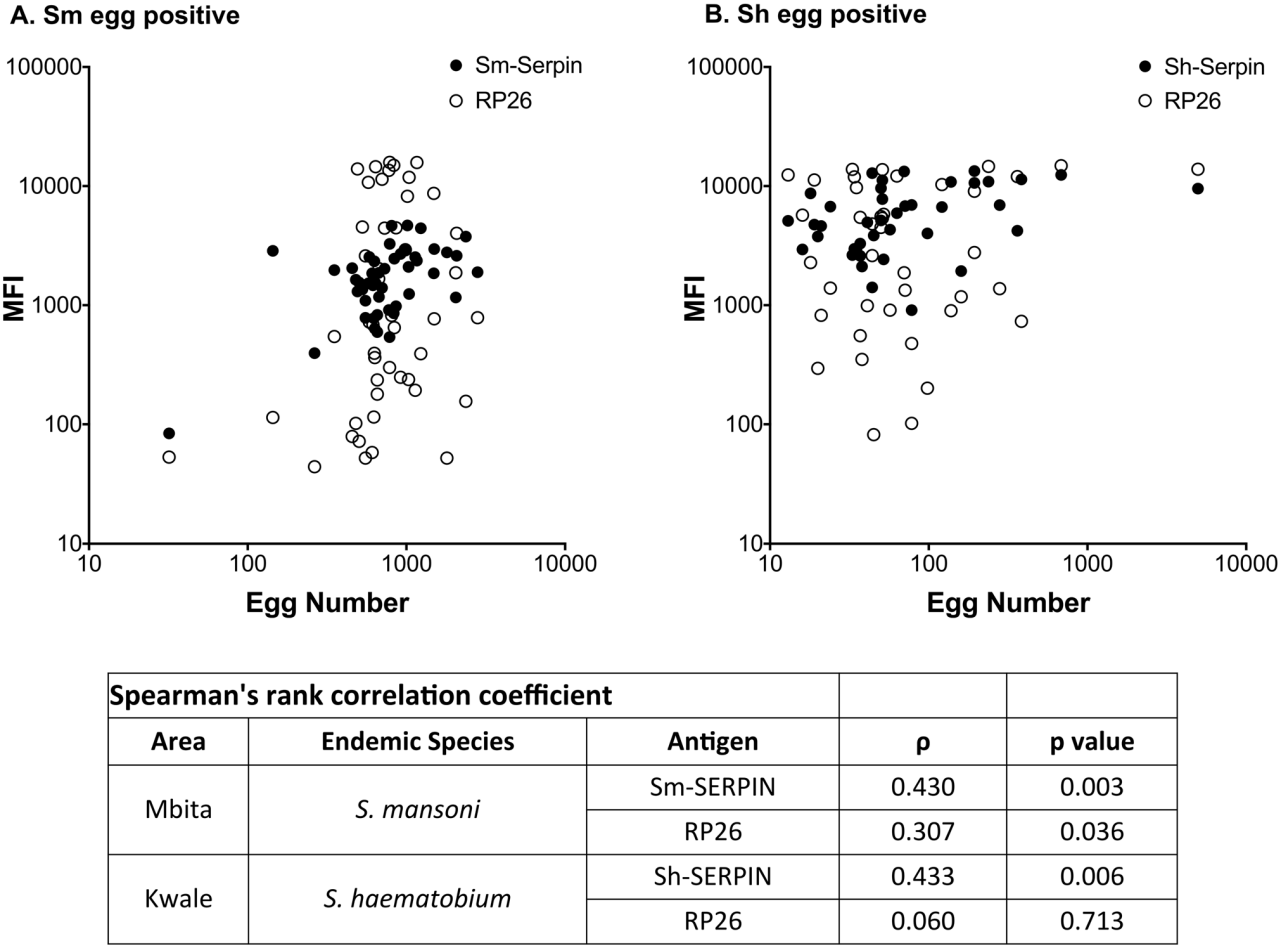
Correlation analysis of the MFI values from immunoassays and egg number. Spearman’s rank correlation coefficient between observed egg number and MFI values were calculated for each antigen. (A) Sm egg positive subjects and (B) Sh egg positive subjects. While the antigens showed poor quantitative correlation with egg number, the SERPINs showed relatively better correlation with egg number than RP26.

The three promising diagnostic antigens from this study will be more valuable if they can also, in addition to qualitative diagnosis, provide an estimate of the number of excreted eggs. Such information will be very useful in planning interventions and for prioritization of intervention to the high-risk populations. Spearman’s rank correlation coefficient was applied for identification of correlation between MFI signals from multiplex immunoassays and the number of excreted eggs from subjects (Fig 7). The Spearman’s rank correlation coefficient showed that the multiplex assay for Sm-SERPIN and Sh-SERPIN had a statistically significant correlation to average number of observed eggs (for Sm-SERPIN, ρ (rho) = 0.430, p-value = 0.003; for Sh-SERPIN, ρ = 0.433, p-value = 0.006). Although Sm-RP26 could detect both species in the earlier described immunoassays, it only showed slight correlation with only *S*. *mansoni* egg number (ρ = 0.307, p-value = 0.036), but not for *S*. *haematobium* (ρ = 0.060, p-value = 0.713). Taken together, these data indicate the diagnostic potentials of Sm-SERPIN and Sh-SERPIN for respective species, and strongly support their inclusion in the multiplex immunoassay system.

## Discussion

The World Health Organization and schistosomiasis researchers have finally made the decision to move from morbidity control to attempt elimination when feasible. Driven by the success of mobilization and donation of PZQ by pharmaceutical companies, the World Health Assembly (WHA) in its most recent resolution (WHA 65.21 of 2012) have provided fresh impetus and political will towards the elimination of schistosomiasis [5, 28]. The major tool for this elimination attempt is MDA using PZQ. This “single-pronged” approach is very worrisome due to the risk of emergence of schistosomes with sub-optimal response to PZQ [4, 28]. This makes it imperative that the MDA programme is carefully planned, implemented, monitored and evaluated, with effective diagnosis as the mainstay of this approach. Effective diagnosis is required for disease mapping, an imperative for schistosomiasis MDA implementation [29]. There is also need for sufficiently sensitive and specific diagnostics for monitoring progress of the intervention and for disease surveillance post- elimination [5, 30]. The Kato-Katz stool examination is still the gold standard due to its high specificity, although less sensitive [6]. Alternative less cumbersome methods have been proposed; especially the point-of care circulating cathodic antigen (POC-CCA) [31, 32]. This method has shown significantly improved sensitivity, albeit with low specificity [7, 9] relative to Kato-Katz. This situation has even led to arguments for combination of methods [8], which further emphasizes the need for continued search for a diagnostics with the desired levels of sensitivity and specificity.

Because several other NTDs and other tropical infectious diseases can also be controlled using the MDA approach, the integration of their control or elimination has become the preferred approach [10]. This is particularly relevant given the limited human and material resources for health intervention in the affected populations. This led us to apply the novel multiplex technology for simultaneous diagnosis of several diseases, reducing the requirement for manpower for single-disease approach and the cost of disease-specific diagnostic kits [12]. The multiplex approach is also amenable for embedding into the health system in developing countries as it can be designed to be site-specific, depending on the prevalent pathogens in each locality. Here, in order to select an appropriate *Schistosoma* antigen for inclusion in the multiplex immunoassay, we assessed a set of previously identified diagnostic antigens. We found that Sm-RP26 and SERPINs are suitable for inclusion in this multi-pathogen surveillance tool. The SERPIN antigens showed sensitivity levels higher than the gold standard, with specificity levels comparable to that of the widely successful POC-CCA [7, 32, 33].

We further found that Sm-SERPIN was able to effectively differentiate species of the parasite. Unexpectedly, our results showed cross-reactivity of Sh-SERPIN to *S*. *mansoni* infected patients, although reactivity was higher to *S*. *haematobium* infected patients than to *S*. *mansoni*. In multiplex assay, because both SERPINs can be included in same assay panel, species-specific Sm-SERPIN will point *S*. *mansoni* infected patients out in endemic area of single species. But this cross-reactivity will be problem for surveillance in area where both strains are endemic. One of possible approach to get better specificity by SERPINs is finding species-specific epitope in SERPINs. The species-specific epitope will be located in unique sequence of each of the SERPINs (Fig 4). We are presently working towards this direction. This species-specific characteristic is advantageous for planning surveys and post-elimination surveillance, in addition to its utility during drug and vaccine development and for planning other non-chemotherapy based interventions [34].

Another significant challenge in schistosomiasis diagnosis is the absence of a true quantitative gold standard with correlation with actual egg burden, apart from Kato-Katz [9]. In this study, we found correlation between the multiplex immunoassay MFI signals and the number of excreted eggs. The MFI is a quantitative unit that lends itself to optimization for quantitative assessment of disease burden, although further extensive studies are still required to establish the utility of these antigens in quantitative assessment of disease burden.

The immunodiagnostic potentials of the SERPINs stems from the important physiological roles they play in schistosomes-host interactions [35, 36]. Furthermore, the presence of multiple copies of the gene encoding *Schistosoma* SERPIN emphasizes its essential role in parasite adaptation in the host milieu [37]. SERPINs have been shown to be highly immunogenic, showing strong reactivity to sera from infected rat [35], sera from rabbit vaccinated with UV-attenuated schistosomula [38], and sera from a naturally resistant host, *Microtus fortis* [39]. The unusually low immunogenicity of helminths serine proteases is attributed to their tight interaction with SERPINs [40]. SERPINs bind tightly and form complexes with otherwise immunologically active proteases, rendering them immunologically inert [40, 41]. These molecular interactions form the basis of SERPINs immunomodulatory and immune evasion roles in the host-parasite interaction [35, 41, 42]. *Schistosoma* SERPINs are expressed in all stages of the parasite [35] as secreted or tegument localized proteins [43]. This localization at the host-parasite interface and inhibitory interaction with host immune effector proteases further explain the immunogenicity, immunomodulatory and immune-evasion roles of schistosomes SERPINs.

We also identified the *Schistosoma* recombinant protein RP26 (Sm22.3) as a promising diagnostic candidate for inclusion in the multiplex immunoassay. The fact that this antigen is able to detect both species of schistosomes may be useful and cost-effective when considered from the point of view of diagnosing all species of schistosomes using one kit [44]. RP26 has also been shown to be valuable in identifying acute infections [24, 45]. Indeed, RP26 is expressed at the cercarial, schistosomula and immature adult stages of the parasite, and not in the egg stage [45]. It was found that mean IgG reactivity to RP26 was significantly higher during acute schistosomiasis, compared to the negative reaction to sera from chronic infection [24, 45]. This suggests that the RP26 antigen is mainly exposed to the host immune response at the acute stage of the infection. This is an interesting phenomenon because IgG response against most parasite antigens requires longer time to develop and rarely manifests at the early stage of the infection, relative to other isotypes [46, 47]. This will be particularly useful in monitoring progress of interventions by detecting new infections.

In summary, a set of previously published promising diagnostic antigens of *S*. *mansoni* were evaluated for suitability for inclusion in our multiplex disease surveillance system. Out of 10 selected antigens, Sm-SERPIN and Sm-RP26 showed significantly high reactivity to sera from schistosomiasis patients and good diagnostic potential. While Sm-RP26 could diagnose both species of the parasites endemic in the study areas, Sm-SERPIN was species-specific. Sh-SERPIN was partially cross-reactive to *S*. *mansoni* infected patients. The advantage of species-specific diagnosis by Sm-SERPIN and its potential diagnostic application in a multiplex format is described taking into account its role in several physiological processes. Sm-RP26 detection of both species is discussed in terms of its previously reported ability to specifically detect acute infection, and its potential utility in monitoring progress of intervention and post-elimination surveillance.

### Accession number for proteins

GeneBank accession number for each antigen were as follows; Cathepsin B(Sm31): AAA29865, Filamin: CCD59036, GAPDH: P20287, GST (Sm28): P09792, LAP-1: ACQ77148, LAP-2: ACQ77149, Legumain (Sm32): P09841, RP26 (Sm22.3): AAB81008, Sm-Serpin: CCD60071, Tropomyosin-2: P42638, Sh-Serpin: AAA19730.
